# Work stress, fatigue and risk behaviors at the wheel: Data to assess the association between psychosocial work factors and risky driving on Bus Rapid Transit drivers

**DOI:** 10.1016/j.dib.2017.09.032

**Published:** 2017-09-25

**Authors:** Sergio Useche, Boris Cendales, Viviola Gómez

**Affiliations:** a University of Valencia, Valencia, Spain; b El Bosque University, Bogotá, Colombia; c University of Los Andes, Bogotá, Colombia

**Keywords:** Professional drivers, Work stress, Fatigue, Psychological distress, Risk behaviors, Bus Rapid Transport, BRT

## Abstract

This Data in Brief (DiB) article presents a hierarchical multiple linear regression model that examine the associations between psychosocial work factors and risk behaviors at the wheel in Bus Rapid Transit (BRT) drivers (n=524). The data were collected using a structured self-administrable questionnaire made of measurements of wok stress (job strain and effort- reward imbalance), fatigue (need for recovery and chronic fatigue), psychological distress and demographics (professional driving experience, hours driven per day and days working per week). The data contains 4 parts: descriptive statistics, bivariate correlations between the study variables and a regression model predicting risk behaviors at the wheel and the entire study dataset. For further information, it is convenient to read the full article entitled “*Stress-related Psychosocial Factors at Work, Fatigue, and Risky Driving Behavior in Bus Rapid Transport (BRT) Drivers*”, published in Accident Analysis & Prevention.

**Specifications Table**

**Table:** 

Subject area	*Psychology*
More specific subject area	*Occupational psychology, risk management, and road safety in the field of public transportation.*
Type of data	*Tables, graph, database*
How data was acquired	*Original data collection*
Data format	*Filtered and Analyzed*
Data source location	*Bogotá, Colombia*
Data accessibility	*Presented data is derived from the original database reported in the article. It also contains the full database obtained for the study,*

**Value of the data**

• This data provides information on the psychosocial work factors associated with risk behaviors at the wheel in BRT drivers.
• The data on the psychosocial work factors of BRT drivers can be compared with those of other groups of professional drivers.
• The data could be generalized to other BRT-based transport systems (BRT systems exist in more than 160 cities in 33 countries).
• The data can be used by other researchers to analyze the working conditions of BRT drivers.

## 1 Design, materials and methods*

### 1.1 Participants

In this cross-sectional study, the sample was made up of 524 male Bus Rapid Transit (BRT) operators from companies affiliated to the Transmilenio S.A. mass transport system in Bogota, Colombia. The mean age of professional drivers was of 40.6 years (SD=7.6) [20–65 range] and average driving experience was of 17.6 years (SD=7.3).

### 1.2 Questionnaire

The Job Content Questionnaire (JCQ) [4], [5] was used for the measurement of job strain and social support. The Effort/Reward Imbalance (ERI) Questionnaire [7], [8], [3] was used for the measurement of the occupational effort-rewards imbalance. The Checklist Individual Strength [12] and Need for Recovery after Work Scale [9], [10] were used respectively to assess fatigue and the need for recovery. Psychological distress was measured using the General Health Questionnaire (GHQ-12. [2]). Finally, risk behaviors at the wheel were measured using a 21-item adapted version for BRT drivers of the Driving Behavior Questionnaire (DBQ) [1], [6].

^*^For further information, please refer to Useche, Cendales and Gómez [3], [11].

### 1.3 Statistical analysis

Hierarchical linear regressions were used to examine the effect of the psychosocial work factors on the risk behaviors at the wheel. The “job strain score” was calculated through the ratio between psychological demands and decision latitude scales of the JCQ. Likewise, the effort-rewards imbalance score was calculated through the algorithm E/R*C, where “E” and “R” are the scores on the effort and reward scales of the ERI Questionnaire respectively, and “C” corresponds to the correction factor for the different number of items in the numerator and denominator. Driving experience, hours driven per day and days working per week were introduced in the first step of the regression model. Job strain and social support were included in the second step, effort-reward Imbalance in the third step, need for recovery (job-related fatigue) in the fourth step, general fatigue in the fifth step, and psychological distress in the sixth step.

## 2 Data

**Fig. 1:**
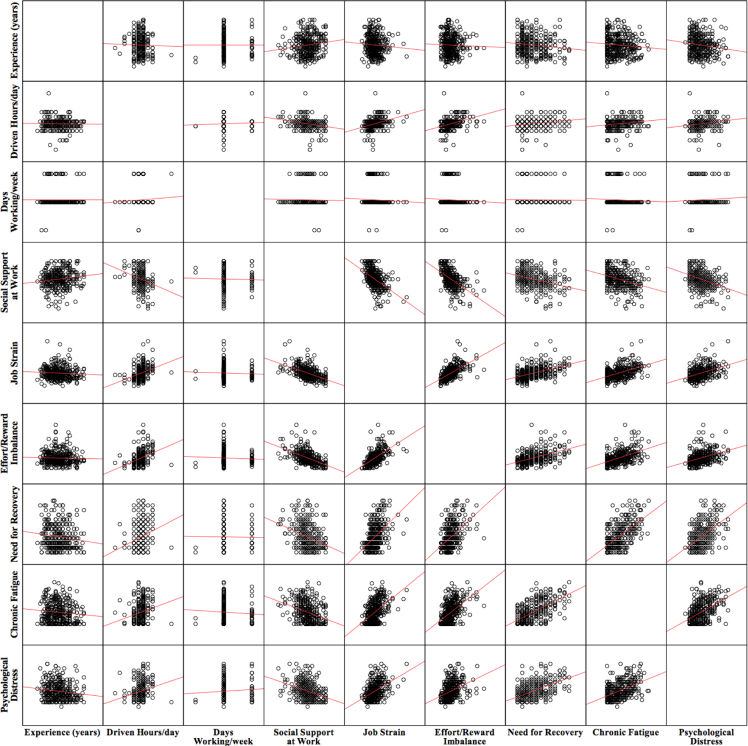
Graphical bivariate correlations between factors included in the dataset.

The dataset of this article provides information on the psychosocial work factors associated with risk behaviors at the wheel on BRT drivers. Table 1. Shows the descriptive statistics. Fig. 1 shows a bivariate Pearson's correlation matrix between the study variables. And Table 2 summarizes the results of a hierarchical linear regression model that examine the associations between psychosocial work factors and risk behaviors at the wheel in BRT drivers. Annex database (.sav) allows to perform additional and specific analyzes using study variables.

**Table 1:** Descriptive statistics of the variables contained in the data set.

**Variable**	**N**	**Minimum**	**Maximum**	**Mean**		**Std. Deviation**
				**Statistic**	**Std. Error**	
Experience (years) as Professional Driver	517	2	43	17,62	0,32	7,31
Driven Hours/day	504	2	14	7,55	0,05	1,11
Days Working/week	511	5	8	6,08	0,01	0,32
Social Support	507	11	32	24,08	0,17	3,84
Job Strain	454	0,26	2,67	0,96	0,02	0,32
Effort/Reward Imbalance	480	0,06	0,74	0,20	0,00	0,06
Need for Recovery	492	0	11	3.138	0,12	2.59
Chronic Fatigue	467	8	45	21.097	0,40	10.08
Psychological Distress	493	13	33	19,95	0,17	3,86

**Table 2:** Hierarchical linear regression model (dependent variable: Risk Behaviors at wheel).

	**Unstandardized Coefficients**		**Standardized Coefficients**	**t**	**Sig.**	**95% Confidence Interval for B**		**∆ R**
	B	Standard Error	Beta			Lower Bound	Upper Bound	
*Step 1*								
Experience (years) as professional driver	-,009	,003	-,165	-3,130	,002	-,015	-,003	,085
Hours driven/day	,078	,019	,224	4,234	,000	,042	,115	
Days working/week	-,121	,069	-,093	-1,751	,081	-,257	,015	
*Step 2*								
Job Strain	,296	,073	,249	4,050	,000	,152	,439	,055
Social Support at Work	,000	,006	,000	-,004	,996	-,012	,012	
*Step 3*								
Effort-Reward Imbalance	1,066	,424	,131	2,515	,012	,232	1,901	,016
Step 4								
Need for Recovery	,040	,008	,269	4,683	,000	,023	,056	,053
*Step 5*								
Cronic Fatigue	,012	,003	,286	4,483	,000	,007	,018	,046
*Step 6*								
Psychological Distress	,028	,006	,281	4,844	,000	,017	,040	,051

R^2^= 0,31; F_(9,331)_= 15.819; p=0.000
